# The Pandemic’s Impact on Patients with Bipolar Affective Disorder in a Non-COVID Medical Unit

**DOI:** 10.1192/j.eurpsy.2022.577

**Published:** 2022-09-01

**Authors:** V. Gheorman, A. Ghițan, Ș. Bușe, F. Militaru, M. Pîrlog, I. Udriștoiu

**Affiliations:** University of Medicine and Pharmacy of Craiova, Psychiatry, Craiova, Romania

**Keywords:** BIPOLAR, noncovid, pandemy, Impact

## Abstract

**Introduction:**

Bipolar disorder or manic-depressive illness is a mental disorder which consists of abnormal and long-lasting changes in a person’s mood, energy, and ability to function. The ongoing COVID-19 pandemic restrictions precipitate the condition of those with bipolar affective disorder.

**Objectives:**

We searched for significant differences before and during the pandemic by analyzing socio-demographic data.

**Methods:**

We carried out a research activity at the I Psychiatry Clinic of the Clinical Hospital of Neuropsychiatry Craiova. We formed two groups of hospitalized patients during 2019 and during 2020, when the pandemic broke out. The inclusion criterion was the presence of bipolar affective disorder as a primary diagnosis.

**Results:**

The number of cases and the total number of hospitalization days was higher during the pandemic, 101 cases versus 94 cases, 1667 days versus 1184 days. We identified a predominance of females during the pandemic, whereas in the previous year the distribution by sex was approximately equal. Regarding environment, the number of patients from urban and rural areas was approximately equal in 2019, while during the pandemic those in urban areas predominated, possibly due to easier access to psychiatric services. The ages of patients maintained a Gaussian distribution with a concentration of cases between 35-55 years.

**Conclusions:**

While other psychiatric disorders were less present in the clinic during the pandemic, the number of bipolar affective disorder cases increased. Bipolar affective disorder is a major challenge due to the wide range of symptoms which cross with comorbidities that increase the likelihood of a SARS-CoV-2 infection.

**Disclosure:**

No significant relationships.

